# GAPGOM—an R package for gene annotation prediction using GO Metrics

**DOI:** 10.1186/s13104-021-05580-1

**Published:** 2021-04-30

**Authors:** Casper van Mourik, Rezvan Ehsani, Finn Drabløs

**Affiliations:** 1 Department of Cancer Research and Molecular Medicine, NTNU-Norwegian University of Science and Technology, 7491 Trondheim, Norway; 2 Institute for Life Science & Technology, Hanze University of Applied Sciences, 9747 AS Groningen, The Netherlands; 3 Department of Mathematics, University of Zabol, Zabol, Iran; 4 Department of Bioinformatics, University of Zabol, Zabol, Iran; 5 Present Address: Department of Informatics, University of Bergen, 5020 Bergen, Norway

**Keywords:** Gene ontology, Annotation, Prediction, Long non-coding RNAs

## Abstract

**Objective:**

Properties of gene products can be described or annotated with Gene Ontology (GO) terms. But for many genes we have limited information about their products, for example with respect to function. This is particularly true for long non-coding RNAs (lncRNAs), where the function in most cases is unknown. However, it has been shown that annotation as described by GO terms to some extent can be predicted by enrichment analysis on properties of co-expressed genes.

**Results:**

GAPGOM integrates two relevant algorithms, lncRNA2GOA and TopoICSim, into a user-friendly R package. Here lncRNA2GOA does annotation prediction by co-expression, whereas TopoICSim estimates similarity between GO graphs, which can be used for benchmarking of prediction performance, but also for comparison of GO graphs in general. The package provides an improved implementation of the original tools, with substantial improvements in performance and documentation, unified interfaces, and additional features.

## Introduction

The properties of gene products like proteins or non-protein coding RNAs (ncRNAs) can be described with Gene Ontology (GO) terms as provided by the GO Consortium [1]. GO resources describe a gene product by annotating it with standardized terms organized as a DAG (Directed Acyclic Graph). Such annotation should ideally be based on experimental data. However, the amount of experimental information that is available varies a lot, and for many genes very little is known about their function. This is particularly true for most ncRNAs, including long ncRNAs (lncRNAs). The number of lncRNAs seems to be comparable to the number of protein-coding genes [2], and they are known to be important in several processes of gene regulation [3], but any useful annotation is in most cases missing. However, it has been shown for example by Jiang et al*.* that annotation by GO terms to some extent can be predicted [4]. A typical approach will identify sets of genes with correlated expression pattern across several experiments, based on the assumption that genes with correlated expression pattern may be involved in similar processes. It will then use enrichment analysis of GO terms for the gene set to identify these processes. This approach was recently benchmarked using an improved strategy for comparing known and predicted GO terms for genes with known annotation [5]. Although the benchmarking had to be done on protein-coding genes (due to the lack of annotated lncRNA genes), the results indicate that the same approach can be used also on other classes of genes, like lncRNAs.

Here we present a user-friendly and well-documented implementation of two tools for annotation prediction and benchmarking, lncRNA2GOA (lncRNA to GO Annotation) [5] and TopoICSim (Topological Information Content Similarity) [6], integrated into the R package GAPGOM (Gene Annotation Prediction using GO Metrics). This package provides a workbench for exploring annotation prediction. We also show how these tools can be the basis for alternative approaches, by using other types of annotation terms or in combination with GO-based definition of gene sets for enrichment analysis.

## Main text

### Overview

**Fig. 1:**
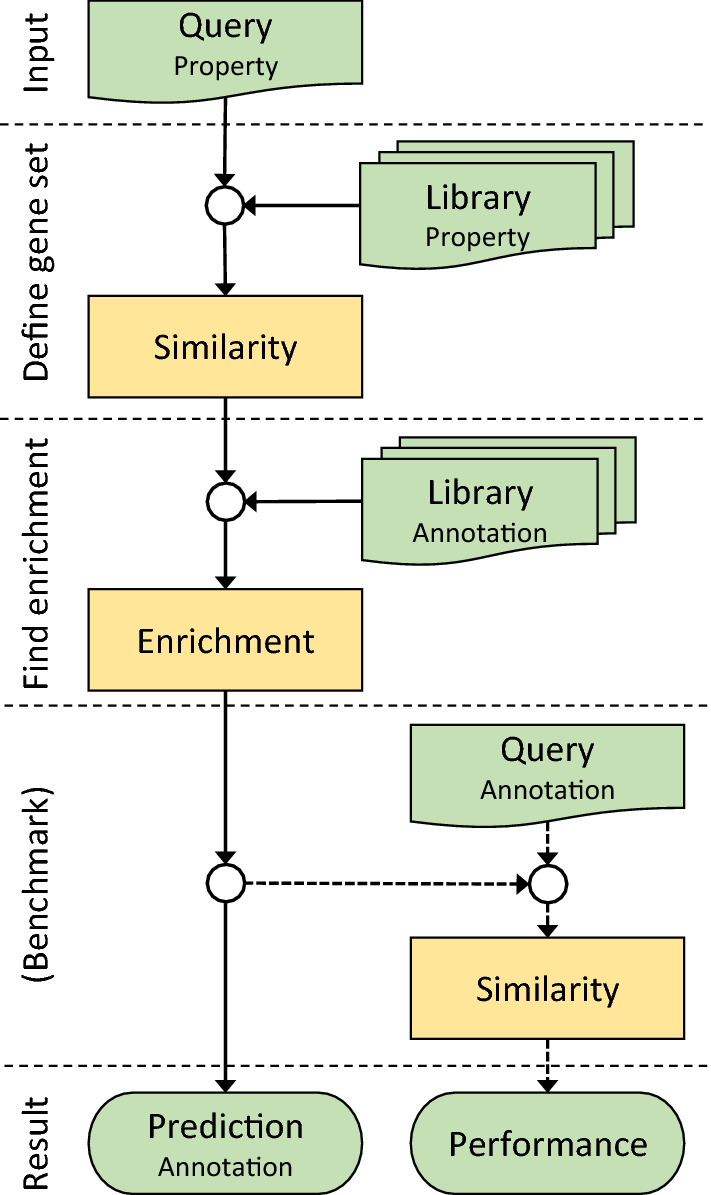
The GAPGOM pipeline for annotation prediction. The flowchart shows the main steps of GAPGOM. For a query gene and a certain property type, a gene set is defined based on similarity over a property library. The gene set is then populated with data from an annotation library and enriched terms of the gene set are identified. The predicted annotation may optionally be compared to actual annotation if the query gene is part of a benchmark dataset (i.e*.*, with known annotation)

GAPGOM integrates the tools lncRNA2GOA and TopoICSim together with libraries on properties like gene expression and annotation data into a pipeline as illustrated in Fig. 1. The main use of GAPGOM will be to go from expression data for a query gene to predicted GO annotation using lncRNA2GOA, by first defining a set of co-expressed genes by correlating the expression of the query gene to other genes across several experiments. The available methods for correlation represent both statistical (Pearson, Spearman, Kendall) and geometrical (Fisher, Sobolev) measures. The tool will then do an enrichment analysis on GO terms for the most correlated genes, by default using the top 250 genes, which previously has been found to be the optimal cutoff [5]. The enriched terms are used as predicted annotation for the query gene. The predicted GO annotation can optionally be compared to actual GO annotation on benchmark sets by using TopoICSim, for example in a simple leave-one-out approach. The overall performance will be indicative of the level of performance that can be expected also for novel genes. Both alternative similarity measures and more advanced cross-validations can easily be integrated with GAPGOM through the R framework.

The lncRNA2GOA tool can also be used in alternative approaches as indicated in Table 1. The main approach described above is Approach 1. A similar approach can be used for predicting “class” rather than GO annotation (Approach 2). The “class” can be any type of annotation term for which we have good training data, as for example whether the gene may be associated with specific types of cancer. This is basically the same as the standard approach, except that we need annotation of “class” rather than GO for the training data. Here we do not need TopoICSim for benchmarking as it will not involve comparisons between complex graph structures of GO terms. Instead, we will typically use a 2 × 2 confusion matrix where predictions on the benchmark set are classified as true or false positive or negative predictions.

**Table 1:** Main alternative approaches for annotation prediction with GAPGOM tools

Approach	Tool	Property	Annotation	Benchmark^a^
1	lncRNA2GOA	Expression	GO	TopoICSim
2	lncRNA2GOA	Expression	Class	2 × 2
3	TopoICSim	GO	Class	2 × 2

^a^2 × 2 represents benchmarking using a confusion matrix over true and false positive and negative predictions

The TopoICSim tool can also be used in another alternative approach (Approach 3) by predicting “class” from gene sets defined through similarity of GO terms rather than gene expression. This means that we can use GO annotation to predict “class”, which can be more specific and informative regarding the property we are investigating, compared to a set of GO terms. Again, benchmark scoring will typically be based on a 2 × 2 confusion matrix.

The use of lncRNA2GOA and TopoICSim has been integrated into the R package GAPGOM, with improved usability and user-friendliness, function documentation and vignettes, significant speed improvements, a more unified input interface, unit tests, and extra features. The main speedup is seen in TopoICSim with more efficient use of R-specific data structures and improvements in the algorithm. Integration through R also makes it easy to add new features. The package is available on Bioconductor and GitHub. Documentation and vignettes are made available in Rmarkdown and HTML (webpage) form and includes both an extensive introduction to the package (An introduction to GAPGOM) and examples of benchmarking (Benchmarks and other GO similarity methods).

### Applications

#### Using co-expression for prediction of GO annotation (approach 1)

The use of lncRNA2GOA for annotation prediction by co-expression in combination with TopoICSim for comparison of GO annotations as an approach to benchmarking has been presented previously [5], although the actual benchmarking had to be based on mRNA data, due to the lack of libraries of well-annotated lncRNAs. We will here just exemplify this approach with a simple benchmark that is available as one of the package vignettes. Here the GSE63733 [7] expression set is used and the annotation of each of the top 10 most variant genes from the Glycolysis hallmark gene set from MSigDB [8] is predicted with lncRNA2GOA. The predicted annotations are compared to the original ones by using TopoICSim. The results demonstrate that the prediction performance on these genes depends upon the ontology. It is best for the Cellular Component ontology (CC) (average similarity of 0.859), followed by Biological Process (BP) (average of 0.699) and Molecular Function (MF) (average of 0.608). A similarity of 1 means that the predicted and actual annotations are identical, whereas 0 means that the two annotations have nothing in common.

#### Using co-expression for prediction of gene class (approach 2)

The approach used in lncRNA2GOA for identifying GO annotation terms through co-expression can in principle be used also on other sources of annotation. A contribution to the LncRNA Function Prediction Challenge at the BIBM 2019 LncRNA Workshop may be used as an example [9]. The organizers of the prediction challenge provided a set of 395 lncRNA genes and their association with 33 different cancer types. Most genes (58%) were associated with just one type of cancer, but the remaining genes were associated with up to 25 different cancer types. Also, the number of lncRNA genes associated with each cancer type varied a lot, from just one gene (like for bladder and testis) to more than 100 different lncRNA genes (colon, lung, liver). The challenge was to use this as a training set to predict cancer association for a set of novel lncRNA genes.

In our first approach to the challenge (the “direct” approach in [9]) the different cancer types were used as annotation terms for the lncRNAs in the training set. For benchmarking each lncRNA was then “re-annotated” with lncRNA2GOA by identifying co-expressed lncRNA genes of the training set and doing enrichment analysis on cancer type for the co-expressed set. This gave a statistically significant enrichment for the expected cancer type, but the enrichment was not very strong. This probably indicates that cancer type may not be strongly associated with co-expression in normal cells.

#### Using GO similarity for prediction of gene class (approach 3)

Here an alternative approach for the prediction challenge was used (the “indirect” approach in [9]), assuming that cancer association could be more directly linked to the function of genes rather than co-expression. Therefore, gene sets were defined by clustering on GO terms, rather than on gene expression. This approach was complicated by the fact that we did not have any GO annotation for the training set. Therefore, the GO annotation of the lncRNAs of the training set was first predicted with lncRNA2GOA. For benchmarking each lncRNA of the training set was then “re-annotated” to cancer type by using TopoICSim to find the other genes of the training set with the most similar set of predicted GO terms and doing enrichment analysis on cancer type for this set. This gave a clearly improved performance, for example when using BP GO terms. The Mathews Correlation Coefficient went from 0.058 for the “direct” approach to 0.514 for the “indirect” approach, and although both prediction results are statistically significant, the Χ^2^ test score (with Yates correction) went from 22.0 for the “direct” approach to 479.2 for the “indirect” approach. Therefore the “indirect” approach represents a very clear improvement in benchmarking results, even though the “indirect” approach uses two stages of prediction, first on GO terms and then on gene class. This example shows the advantage of being able to use both lncRNA2GOA and TopoICSim in combination.

## Discussion and conclusion

The main goal of GAPGOM has been to provide access to lncRNA2GOA and TopoICSim as user-friendly and well-documented tools for doing experiments on annotation prediction by correlation analysis. This is particularly relevant for gene types where a large fraction of the genes may lack functional annotation, like lncRNA genes, but can in principle be used on any expressed gene.

The lncRNA2GOA tool includes the novel correlation metrics Fisher and Sobolev, which can give improved performance [5]. TopoICSim makes it possible to benchmark performance on suitable benchmark sets by comparing predicted and original annotation for all genes in the set, and R as a framework facilitates the use of other metrics that can be made available in R. Users can thereby evaluate the effect of alternative settings for annotation prediction, like alternative libraries of expression data. During GAPGOM development expression data from both FANTOM 5 [10] and selected TCGA [11] datasets have been used.

There are other tools for correlation-based annotation, like LncRNA2Function [4], Co-LncRNA [12], FuncPred [13], lncFunTK [14] or NeuraNetL2GO [15], but many of these tools may be difficult to install and run. As part of a Bioconductor package the lncRNA2GOA tool should install easily on any compatible R system.

The results described here are mainly meant to indicate how GAPGOM can be used, as full evaluation of the performance of lncRNA2GOA and TopoICSim has been documented previously [5, 6]. However, the results presented here show that interesting observations can be made even from relatively simple experiments. The first benchmarking (co-expression to GO annotation) indicates that cellular component (CC) is more directly linked to co-expression compared to MF and BP. The second benchmarking (co-expression to gene class) shows that although there is a significant enrichment for the expected cancer type, the enrichment is not very strong, possibly because gene expression in normal cells may not be a good indicator for possible association with cancer. The third benchmarking (GO similarity to gene class) shows that the best prediction performance is for biological process (BP), which seems reasonable since we are trying to identify genes associated with the process of cancer. In the prediction of cancer genes up to 41% of the predictions were classified as false positive. However, it is possible that some of these false positive predictions are true positive. It may be difficult to define if gene is involved or not in a specific cancer type, and some of the false positive predictions may represent unknown positive cases. However, in all examples more extensive benchmarking can easily be implemented and explored with GAPGOM.

## Limitations

The performance of GAPGOM is mainly limited by the access to expression data and quality of annotation in reference libraries.

## Data Availability

Project name: GAPGOM. Project home page: GAPGOM is available on Bioconductor: https://doi.org/10.18129/B9.bioc.GAPGOM and on GitHub: https://github.com/Berghopper/GAPGOM. Operating system: Platform independent. Programming language: R. Other requirements: None. License: GPLv3 – GNU General Public License (GPL) version 3. Any restriction to use by non-academics: None. The relevant files are included with the Bioconductor package.

## Abbreviations

GO: Gene ontology
CC: Cellular component
BP: Biological process
MF: Molecular function
